# Protective effect of diet supplemented with rice prolamin extract against DNCB-induced atopic dermatitis in BALB/c mice

**DOI:** 10.1186/s12906-015-0892-0

**Published:** 2015-10-14

**Authors:** Hyun-Joong Yoon, Mi-Sun Jang, Hyun-Woo Kim, Dong-Up Song, Kwang-Il Nam, Choon-Sang Bae, Seong-Jin Kim, Seung-Rock Lee, Chang-Sub Ku, Dong-Il Jang, Bong-Whan Ahn

**Affiliations:** Chonnam University Research Institute of Medical Sciences, Chonnam National University, Gwangju, South Korea; Research Center for Oral Disease Regulation of the Aged (MRC), College of Dentistry, Chosun University, Gwangju, South Korea; COTDE Inc. 19-3, Ugakgol-gil, Susin-myeon, Cheonan-si, Chungcheongnam-do 330-882 South Korea

**Keywords:** Dietary rice prolamin extract, Atopic dermatitis, Interferon-gamma

## Abstract

**Background:**

Rice prolamin has been reported to possess antioxidative, anti-inflammatory and immune-promoting properties. This study is aimed to examine the protective effects of dietary rice prolamin extract (RPE) against dinitrochlorobenzene (DNCB)-induced atopic dermatitis (AD)-like skin lesions in mice.

**Methods:**

BALB/c mice were fed diet supplemented with 0–0.1 % RPE for 6 weeks. For the last 2 weeks, 1 % or 0.2 % DNCB was applied repeatedly to the back skin of mice to induce AD-like lesions. Following AD induction, the severity of skin lesions was examined macroscopically and histologically. In addition, the serum levels of IgE, IgG1 and IgG2a were determined by ELISA, and the mRNA expression of IL-4 and IFN-γ in the skin was determined by real-time PCR.

**Results:**

Dietary RPE suppressed the clinical symptoms of DNCB-induced dermatitis as well as its associated histopathological changes such as epidermal hyperplasia and infiltration of mast cells and eosinophils in the dermis. RPE treatment also suppressed the DNCB-induced increase in transepidermal water loss. Dietary RPE inhibited the DNCB-induced enhancement of serum IgE and IgG1 levels, whereas it increased the serum IgG2a level in DNCB-treated mice. In addition, dietary RPE upregulated the IFN-γ mRNA expression and downregulated the IL-4 mRNA expression in the skin of DNCB-treated mice.

**Conclusions:**

The above results suggest that dietary RPE exerts a protective effect against DNCB-induced AD in mice *via* upregulation of Th1 immunity and that RPE may be useful for the treatment of AD.

## Background

AD is a chronic inflammatory skin disease that often begins in infancy. It causes enormous physical discomfort and imposes huge demands on time and resources [1]. AD is characterized by pruritus and eczematous skin lesions, elevated serum immunoglobulin (Ig) E level, and infiltration of immune cells such as mast cells, eosinophils and lymphocytes in the skin [2, 3]. Th2 cells play a key role in the pathogenesis of AD [4, 5]. They synthesize high levels of IL-4 and other Th2 cytokines, which lead to immunoglobulinemia E, eosinophilia, epidermal thickening and other AD-associated inflammatory changes. Conversely, Th1 cells suppress the Th2 immune responses through production of IFN-γ [6]. Therefore, promotion of Th1 immunity and suppression of Th2 immunity can be an effective therapeutic measure for AD [6, 7]. In fact, some strains of probiotic bacteria have been reported to exert beneficial effects in AD by promoting IFN-γ production [8–11].

Rice is a staple food worldwide [12]. In addition, rice has been described to have various pharmacological and biological activities including hypocholesterolemic and anticarcinogenic effects [13–20]. Rice proteins are nutritious for humans with hypoallergenic properties among the cereal proteins. They consist of four important fractions, identified by differential solubility: water-soluble albumin, salt-soluble globulin, alkali-soluble glutelin and alcohol-soluble prolamin [12, 21]. Recently, antioxidative [22], anti-inflammatory [23] and immune-promoting [24] activities of rice prolamin have been reported. According to Chen et al. and other investigators [24, 25], human peripheral blood mononuclear cells (HPBMCs) exposed to rice prolamin secreted IFN-γ, and the conditioned medium prepared from HPBMCs cultured in the presence of rice prolamin inhibited the growth of human leukemia U937cells and triggered the differentiation of the cells toward monocytes. Because rice prolamin is indigestible [12, 26], an exception among the rice proteins, dietary rice prolamin seems to pass through the intestine in macromolecular forms, where it may induce immune reactions. Therefore, the above observations suggest that dietary rice prolamin may be beneficial for AD *via* immunomodulatory function.

In the present study, we examined the protective effects of dietary supplementation of rice prolamin extract (RPE) on DNCB-induced AD-like lesions in BALB/c mice. We also examined the effects of dietary RPE on Th1 and Th2 immunities in the above mice.

## Methods

### Animals and materials

Six-week-old female BALB/c mice were purchased from Jungang Lab Animal, Inc. (Seoul, Korea) and were housed in an air-conditioned room (22 ± 2 °C) with a 12-h dark–light cycle and were allowed free access to water and food. 2, 4-Dinitrochlorobenzene (DNCB) was purchased from Sigma-Aldrich Co. (St. Louis, MO, USA) and dissolved in acetone-olive oil (4:1, v/v). All other reagents used were of the analytical grade commercially available. This study was approved by the Institutional Animal Care and Use Committee of Chonnam National University Medical School (approval No.: CNU IACUC-H-2015–5).

### Preparation and feeding of rice prolamin extract (RPE)

**Fig. 1:**
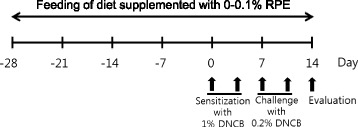
Experimental protocol for rice prolamin extract (RPE) feeding and induction of atopic dermatitis. BALB/c mice were fed diet supplemented with 0, 0.05 % and 0.1 % RPE for 6 weeks. For the last 2 weeks, the back skin of mice was applied repeatedly with DNCB to induce AD. For DNCB sensitization, a 1 cm^2^ gauze-attached patch was applied with 0.1 ml of 1 % DNCB and attached to the back skin for 2 days on day 0 and 3. And for the following challenge process, the gauze-attached patch was applied with 0.1 ml of 0.2 % DNCB and attached to the sensitized area for a day on day 7 and 10. The mice were sacrificed on day 14 to evaluate the effects of DNCB and RPE treatments

Rice (cultivar Dongjinchal) supplied from Changpyeong NH (Damyang, Korea) was ground to pass through a 0.18 mm screen (Daehwa Precesion, Cheonan, Korea). Prolamins were extracted from rice flour with 70 % ethanol, as described previously [27] with minor modifications. Rice flour (100 g) was defatted with hexane and dried in a fume hood at room temperature for 24 h. The defatted flour was then extracted by stirring in 400 ml of 5 % NaCl at 20 °C for 4 h and centrifuged at 3,000 × g for 30 min to remove the albumin and globulin fractions. The residue after extraction of albumin-globulin was extracted with 300 ml of 70 % ethanol at 20 °C for 4 h to isolate the prolamin fraction. Each extraction step was repeated twice to remove most of the protein fraction. The prolamin fraction was then dialyzed against distilled water, lyophilized and then stored at 4 °C. An aliquot of the freeze-dried extract was dissolved in 70 % ethanol containing 25 mM NaOH and its protein concentration was determined with a BCA protein assay kit (Thermo Scientific, Rockford, IL, USA) and purified 13 kDa prolamin as the standard. The average total solids in the prolamin fraction extracted from 100 g rice flour were 0.53 g and the average protein content of the solids was 64.5 %. The mice were fed a dietary powder supplemented with 0, 0.05 % and 0.1 % lyophilized RPE. The dietary powder was prepared by pulverizing the standard laboratory chow R03 (SAFE Lab Diets, Augy, France), and fresh diet was provided daily for 6 weeks according to the schedule summarized in Fig. 1.

### Induction of AD-like lesions

The DNCB patched model described by Lee et al. [28] was used. The backs of mice were shaved with an electric clipper and depilatory cream a day before DNCB sensitization. The DNCB sensitization and challenge were performed for 2 weeks according to the schedule summarized in Fig. 1. For the sensitization process, a 1 cm^2^ gauze-attached patch (Tegaderm®, 3 M Health Care, St. Paul, MN, USA) was applied with 0.1 ml of 1 % DNCB and attached to the shaved area for 2 days on day 0 and 3. For the challenge process, the gauze-attached patch was applied with 0.1 ml of 0.2 % DNCB and attached to the sensitized area for a day on day 7 and 10. The mice were sacrificed on day 14 to evaluate the effects of DNCB and RPE treatments.

### Assessment of the severity of skin lesions

The severity of DNCB-induced skin lesions was clinically assessed as previously described [29, 30]. The dermatitis score was defined as a sum of individual scores (0, no symptom; 1, mild; 2, moderate; 3, severe) for the following four signs and symptoms: erythema/hemorrhage, edema, erosion and dryness.

### Measurement of transepidermal water loss (TEWL)

TEWL was measured under forane anesthesia using a Tewameter TM300 (Courage and Khazaka Electronic GmbH, Köln, Germany) in a climate-controlled room.

### Histopathological analysis

The dorsal skins of the mice were removed and fixed in 10 % phosphate-buffered formalin. The skin sections (4 μm thick) were stained with hematoxylin and eosin to evaluate the epidermal hyperplasia and the other sections were stained with toluidine blue and Giemsa to evaluate the infiltration of mast cells and eosinophils.

### Measurement of serum immunoglobulins

Blood samples were collected by cardiac puncture under anesthesia, and sera were collected by centrifugation and stored at −80 °C until use. The serum IgE, IgG1 and IgG2a levels were measured with monoclonal antibody pairs by sandwich ELISA using commercial kits (eBioscience, San Diego, CA, USA). Briefly, microtiter plates were coated with monoclonal rat antimouse IgE, IgG1 or IgG2a antibody, followed by sequential incubation of serially diluted purified mouse IgE, IgG1 and IgG2a (standards) or sera (in triplicate), horseradish peroxidase-conjugated monoclonal antimouse IgE, IgG1 or IgG2a antibody, and then the substrate. The absorbance of the resulting product was read using a microplate reader (BioTek Instruments, Inc., Winooski, VT, USA).

### Measurement of IL-4 and IFN-γ mRNA expression in the skin by real-time polymerase chain reaction (RT-PCR)

Total RNA was extracted from skin tissue with the TRIzol reagent (Invitrogen Life Technologies, Grand Island, NY, USA) according to the manufacturer’s instruction. The quantity and purity of total RNA were determined with a Nanodrop reader (Nanodrop Technologies, Wilmington, DE, USA). One microgram of total RNA was converted to the first-strand DNA with Moloney murine leukemia virus (MMLV) reverse transcriptase (Invitrogen Life Technologies) and RNAsin (Takara, Otsu, Shiga, Japan). cDNA was amplified using gene-specific primers and GoTaq® DNA polymerase (Promega, Madison, WI, USA). Primer sequences were as follows: IFN-γ, 5′-GTCAACAACCCACAGGTCCA-3′/5′-ACTCCTTTTCCGCTTCCTGA-3′; IL-4, 5′- CTTCCAAGGTGCTTCGCATA-3′/5′-AAGCCCGAAAGAGTCTCTGC-3′; β-Actin, 5′- CTAGGCACCAGGGTGTGATG-3′/5′-GGGGTACTTCAGGGTCAGGA-3′. β-Actin was used as the internal control.

### Statistical analysis

The results are presented as mean ± SD. The significance of differences of all results was analyzed by one-way analysis of variance (ANOVA) followed by the Scheffe’s test.

## Results

### Effect of dietary RPE on clinical symptoms of DNCB-induced dermatitis in BALB/c mice

**Fig. 2:**
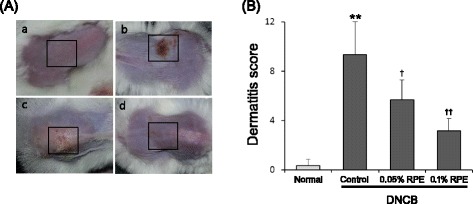
The preventive effect of dietary RPE on AD-like skin lesions induced by DNCB treatment in mice. **a** Photographs showing skin lesions (marked with a square) in the different groups of experimental mice: a, normal control; b, DNCB control; c, DNCB + 0.05 % RPE; d, DNCB + 0.1 % RPE. **b** The severity of clinical symptoms of the skin lesions was evaluated macroscopically and expressed as the dermatitis score (maximum score, 12) which was defined as a sum of individual scores (0, no symptom; 1, mild; 2, moderate; 3, severe) for the following four symptoms: erythema/ hemorrhage, edema, erosion and dryness. **P < 0.01 compared with the normal group, and ^*†*^ *P* < 0.05, ^*††*^ *P* < 0.01 compared with the DNCB control group (*n* = 6)

**Fig. 3:**
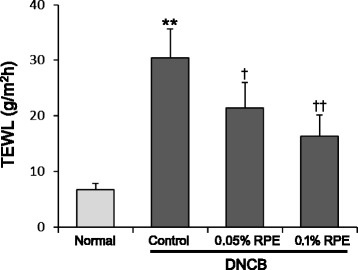
The preventive effect of dietary RPE on DNCB-induced attenuation of skin barrier function in mice, based on transepidemal water loss (TEWL). TEWL was measured using a Tewameter TM300 in a climate-controlled room. **P < 0.01 compared with the normal group, and ^*†*^ *P* < 0.05, ^*††*^ *P* < 0.01 compared with the DNCB control group (*n* = 6)

Following repeated application of DNCB to the back skin of mice for 2 weeks, AD-like lesions developed. Clinical symptoms such as erythema/hemorrhage, edema, erosion and dryness were evident with the average dermatitis score approaching 10 (Fig. 2). Skin barrier function also decreased markedly in DNCB-treated mice compared to the normal group, as evidenced by the increase in TEWL (Fig. 3). Dietary RPE dose-dependently suppressed the clinical symptoms of dermatitis and attenuation of skin barrier function induced by DNCB treatment. It inhibited the DNCB-induced increase in dermatitis score by 40.7 and 68.4 % at 0.05  and 0.1 %, respectively, and inhibited the DNCB-induced TEWL increase by 38.2 and 59.7 % at 0.05  and 0.1 %, respectively (Figs. 2 and 3).

### Histopathological changes in the skin caused by DNCB and RPE treatments

**Fig. 4:**
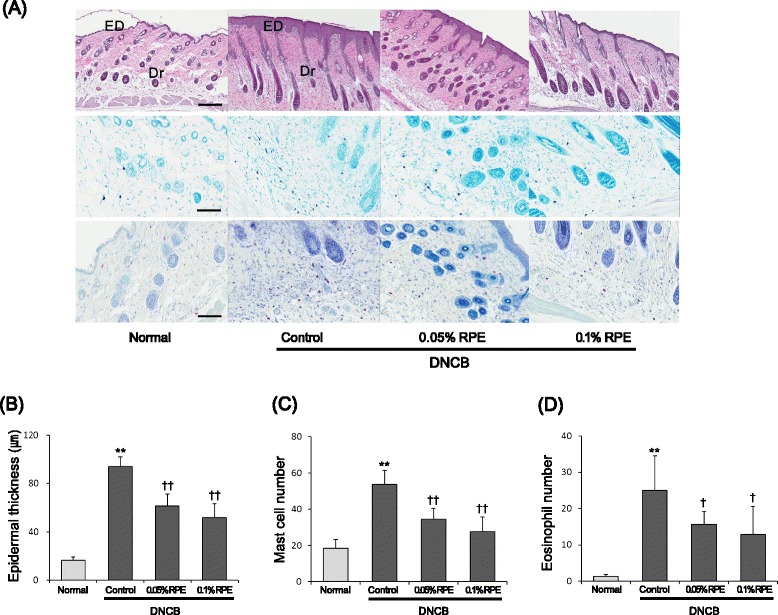
Histopathological findings showing the preventive effect of dietary RPE on DNCB-induced AD-like skin lesions in mice. **a** Skin sections (4 μm thick) were stained with hematoxylin & eosin (upper panel), toluidine blue (middle panel) and Giemsa (lower panel). ED, epidermis; Dr, dermis; magnification, 200 ×; scale bars, 50 μm. **b** Epidermal thickness was analyzed in the hematoxylin & eosin-stained sections. **c** The number of mast cells was analyzed in the toluidine blue-stained sections. **d** The number of eosinophils was analyzed in the Giemsa-stained sections. **P < 0.01 compared with the normal group, and ^*†*^ *P* < 0.05, ^*††*^ *P* < 0.01 compared with the DNCB control group (*n* = 6)

We examined the histopathological changes in the back skins of experimental mice (Fig. 4). The skins of the DNCB-treated group showed markedly epidermal hyperplasia as compared to the normal group. Intriguingly, this hyperplasia was significantly reduced by RPE treatment (Fig. 4a, b). As seen in toluidine blue and Giemsa stains, the number of mast cells and eosinophils in the dermis of DNCB-treated mice was markedly increased as compared to the normal group. In addition, these DNCB-induced increments of inflammatory cells were significantly suppressed by RPE treatment (Fig. 4a, c, d).

### Changes in the serum concentrations of IgE, IgG1 and IgG2a caused by DNCB and RPE treatments

Because serum IgE levels are correlated with the severity of AD and the serum levels of IgE, IgG1 and IgG2a are associated with Th2 or Th1 immunity [1, 31], we examined the serum levels of these three Igs in mice to evaluate the effects of DNCB and RPE on systemic Th1 and Th2 immunities (Table 1). The serum levels of IgE and IgG1 were markedly increased in the DNCB-treated group, although the serum IgG2a level was not changed significantly by this treatment. Dietary RPE influenced the serum Ig levels differently in DNCB-treated mice. It dose-dependently reduced the serum levels of IgE and IgG1, whereas 0.1 % RPE significantly increased the serum IgG2a level in DNCB-treated mice.

**Table 1:** Effects of dietary RPE on serum levels of lgE, lgG1 and lgG2a in DNCB-treated mice

Treatment	lgE (ng/ml)	lgG1 (mg/ml)	lgG2a (mg/ml)
Normal	42.7 ± 14.6	0.334 ± 0.041	0.102 ± 0.019
DNCB	264.0 ± 38.5**	2.326 ± 0.411**	0.110 ± 0.013
DNCB + 0.05 % RPE	175.0 ± 26.9^††^	1.648 ± 0.274^††^	0.124 ± 0.024
DNCB + 0.1 % RPE	148.7 ± 25.4^††^	1.388 ± 0.216^††^	0.159 ± 0.016^†^

The serum lg levels were determined by ELISA. **P <0.01 compared with the normal group, and ^*†*^ *P* <0.05, ^*††*^ *P* <0.01 compared with the DNCB control group (*n* = 6)

### DNCB and RPE treatments change the mRNA expression of IL-4 and IFN-γ in the skin

**Fig. 5:**
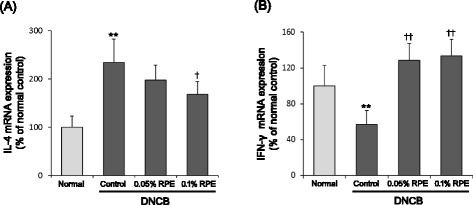
Effects of dietary RPE on mRNA expression of IL-4 (**a**) and IFN-γ (**b**) in the skin of DNCB-treated mice. The cytokine mRNA expressions were determined by real-time PCR. **P < 0.01 compared with the normal group, and ^*†*^ *P* < 0.05, ^*††*^ *P* < 0.01 compared with the DNCB control group (*n* = 6)

To evaluate the effects of DNCB and RPE treatments on skin Th1 and Th2 immunities, we examined the expression of IL-4 and IFN-γ mRNAs in the back skins of experimental mice. As shown in Fig. 5, the IL-4 mRNA expression was markedly increased by DNCB treatment, whereas, the IFN-γ mRNA expression was significantly decreased in DNCB-treated mice as compared to the normal group. These results are consistent with earlier observations [28]. Dietary RPE reversed these DNCB-induced changes in Th1 and Th2 cytokine mRNA expressions. That is, RPE treatment suppressed the DNCB-induced increase of IL-4 mRNA expression (Fig. 5a), whereas it increased the IFN-γ mRNA expression up to more than twice the level of the DNCB control group (Fig. 5b).

## Discussion

This study showed that dietary RPE supplementation inhibited the AD-like pathology induced by DNCB treatment in BALB/c mice. RPE treatment suppressed not only the clinical symptoms of dermatitis such as erythema, edema, erosion and dryness (Fig. 2), but also its histopathological changes such as epidermal hyperplasia and infiltration of mast cells and eosinophils in the dermis (Fig. 4). In addition, RPE treatment suppressed the DNCB-induced attenuation of skin barrier function, as evidenced by the changes in TEWL (Fig. 3). The immune cells infiltrated in the skin tissue, following antigen binding, become activated and secrete a variety of bioactive chemical mediators including histamine, proteases, eicosanoids, cytokines and chemokines, and proinflammatory proteins [32]. Bioactive mediators can cause locally and systemically diverse symptoms and signs including those observed in Figs. 2, 3, and 4 [32]. Therefore, our results indicate that dietary RPE may effectively prevent AD symptoms.

As shown in Table 1, dietary RPE reduced the serum levels of IgE and IgG1, whereas it raised the serum IgG2a level, in DNCB-treated mice. It is known that IFN-γ stimulates the expression of IgG2a and inhibits the production of IgE and IgG1. In contrast, IL-4 has powerful effect in stimulating the expression of IgE and IgG1 but markedly inhibits the expression of IgG2a [31]. Although the mechanism for the anti-AD action of RPE remains unclear, the results of Table 1 strongly suggest that upregulation of Th1 immunity and downregulation of Th2 immunity are a principal mechanism for the RPE action. Indeed, we found that RPE treatment suppressed IL-4 mRNA expression and raised IFN-γ mRNA expression in the skin of DNCB-treated mice (Fig. 5), supporting the above suggestion that upregulation of Th1 immunity and downregulation of Th2 immunity are involved in the anti-AD action mechanism of dietary RPE. Because Th2 cytokine production is a default pathway in many systems [33–35] and Th1 and Th2 expressions are antagonistic to each other, it follows that upregulation of IFN-γ expression by RPE may be the primary causative factor that leads to downregulation of Th2 expression and other RPE effects observed in this study. For example, IFN-γ can seriously influence Th1/Th2 cell differentiation and cytokine production [36]. IFN-γ activates signal transducer and activator of transcription 1 (Stat1), which upregulates the leading Th1 transcription factor, T-bet, further enhancing Th1 cytokine production. Concurrently, IFN-γ inhibits Th2 cytokine production by interfering with GATA, a Th2 transcription factor. Furthermore, inflammatory cells in local skin lesions are recruited by complex interactions of chemokines and chemokine receptors whose expression is also regulated by Th1 and Th2 cytokines [32, 37]. IFN-γ amplifies Th1 responses by inducing Th1-type chemokines and their receptors and by preventing the expression of Th2-type receptor ligands. AD is characterized by hyperactivated Th2 cytokines that lead to immunoglobulinemia E, eosinophilia, epidermal thickening and other AD-associated inflammatory changes [4, 5]. These Th2 immune responses, however, can be suppressed by IFN-γ [6]. The formation and maintenance of skin barrier are also influenced by Th1 and Th2 cytokines [38]. IFN-γ stimulates ceramide synthesis through the action of enzymes, sphingomyelinase and glucocebrosidase, resulting in suppression of TEWL [38, 39]. And IFN-γ may play a role in maintaining the skin barrier by regulating Th2 cytokine receptors, because Th2 cytokines inhibit the formation of skin barrier [38]. Thus, IFN-γ can modify all the DNCB-induced AD-like changes observed in this study: clinical symptoms, attenuation of skin barrier function, immune cell infiltration, and changes in serum IgE, IgG1 and IgG2a levels and skin Th1 and Th2 cytokine mRNA expressions.

Although earlier studies showed that PBMCs exposed to rice prolamin secreted IFN-γ *in vitro* [27, 28], it is uncertain how dietary RPE can activate Th1 responses *in vivo*. As dendritic cells are the only antigen presenting cells known to sample luminal contents from the intestine [35, 40], they play a critical role in Th1 cell activation by commensal bacteria- and food-associated antigens [35]. They sample and uptake antigens from the gastrointestinal luminal compartment and processed the antigens [40–42]. After migrating to nearby lymph nodes, they present the processed antigens to T cells and stimulate T cell differentiation. They also secrete IL-12, which binds to the IL-12 receptor on T cells and signals to activate Th1 cell differentiation [36]. The differentiated Th1 cells can then migrate to extraintestinal sites [35]. It will be an important task to find out whether dietary RPE acts in a similar way to the above mentioned antigens or if it acts by other mechanisms.

In the present study, dietary RPE significantly prevented the AD-like symptoms induced by DNCB treatment in mice and it improved the Th1/Th2 balance that was skewed to Th2 by DNCB treatment. Because rice is a common, stable and safe food, RPE can be a potential resource for the development of new therapeutic agents for AD. Further studies including clinical researches are required to prove this possibility.

## Conclusions

This study indicated that dietary RPE was protective against DNCB-induced AD-like lesions in BALB/c mice. Our results suggest that dietary RPE exerts its anti-AD effect *via* upregulation of Th1 immunity and that RPE may be useful for the treatment of AD.
